# Influence and distinctions of particulate matter exposure across varying etiotypes in chronic obstructive pulmonary disease (COPD) mouse model

**DOI:** 10.1186/s12950-024-00416-8

**Published:** 2024-11-01

**Authors:** Jung Hur, Chin Kook Rhee, Hyoung Kyu Yoon, Chan Kwon Park, Jeong Uk Lim, Tai Joon An, Joon Young Choi, Yong Suk Jo

**Affiliations:** 1 Division of Pulmonary and Critical Care Medicine, Department of Internal Medicine, College of Medicine, Seoul St. Mary’s Hospital, The Catholic University of Korea, Seoul, South Korea; 2 Division of Pulmonary and Critical Care Medicine, Department of Internal Medicine, Yeouido St Mary’s Hospital, College of Medicine, The Catholic University of Korea, Seoul, South Korea; 3 Division of Pulmonary and Critical Care Medicine, Department of Internal Medicine, Incheon St. Mary’s Hospital, College of Medicine, The Catholic University of Korea, Seoul, South Korea

**Keywords:** Asthma, Chronic obstructive pulmonary disease, Particulate matter, Oxidative stress

## Abstract

**Background:**

Air pollution, notably particulate matter (PM), significantly impacts chronic respiratory disease such chronic obstructive pulmonary disease (COPD). Although asthma-COPD overlap (ACO), considered one of the COPD etiotype, is associated with greater severity in both symptoms and outcomes, effects of PM exposure remain unclear. Thus, this study aimed to evaluate impact of PM on chronic airway disease animal models.

**Methods:**

We established two distinct COPD etiotypes, cigarette smoking-related COPD (COPD-C) and COPD with asthma (COPD-A), using porcine pancreatic elastase (PPE) for COPD-C and a combination of PPE with ovalbumin for COPD-A. To reflect smoking influence, cigarette smoking extract was administered to both disease models. To assess impact of PM exposure, bronchoalveolar lavage fluid (BALF), proinflammatory cytokines, lung histology, and cellular damage mechanisms were analyzed.

**Results:**

In the COPD-A model, cell counts and type 2 cytokines were elevated in BALF independent of PM exposure. All models exhibited increased lung inflammation and emphysema due to PM exposure. Expression levels of apoptosis-related protein B-cell lymphoma protein 2 (Bcl-2) associated X (Bax) showed an inclination to increase with PM exposure. In the COPD-A model, decreased expression of basal nuclear factor erythroid-derived 2-like 2 (Nrf-2) and increased production of reactive oxygen species (ROS) due to PM exposure were noted.

**Conclusion:**

We developed two distinct models for the etiotypes of COPD and found increased vulnerability to cell damage in COPD-A after PM exposure. Moreover, the control group displayed escalated airway inflammation and emphysema due to PM exposure, substantiating the risk of respiratory diseases.

**Supplementary Information:**

The online version contains supplementary material available at 10.1186/s12950-024-00416-8.

## Introduction

Air pollution encompassing various pollutants and particulate matter (PM) of varying sizes and composition has gained substantial attention in recent years due to its detrimental impact on human health. Airway is the main entrance of ambient pollutant to the body. Its impact on the development, progression, and exacerbation of chronic airway diseases such as asthma and chronic obstructive disease (COPD) has been well described in previous studies [1–6].

PM, a multifaceted blend of airborne liquid droplets and solid particles, is characterized by its diminutive size, typically under 10 μm in diameter. Notably, these minute particles of PM are capable of infiltrating the finer passageways and alveoli of the respiratory system, posing significant risks to the health of the airways and lungs by directly impacting these areas. Toxic effects of PM are multifaceted, involving oxidative stress, mitochondrial damage, inflammation, apoptosis and autophagy disruption, extracellular matrix remodeling, and dysregulation of various signaling pathways [7].

Asthma is characterized by variable expiratory airflow limitation. COPD is characterized by persistent airflow limitation. Both diseases are not mutually exclusive. They can exist together in the same patient. In patients who have long-standing asthma, persistent airflow limitation might be developed, especially for those with severe asthma. Distinguishing these asthma patients from COPD patients is difficult, especially if they have risk factors for COPD such as smoking and other biomass exposure history [8–11]. On the other hand, some COPD patients exhibit eosinophilic airway inflammation and responses to corticosteroid therapy [11, 12]. These overlapped patients have been called asthma-COPD overlap (ACO) patients. While ACO is not considered a single disease entity, individuals with ACO have garnered significant attention in recent years due to their distinct clinical characteristics. Individuals with ACO experience a more pronounced severity of respiratory symptoms, exacerbations more frequently, confront a diminished quality of life, suffer from reduced pulmonary function, and have an elevated risk of mortality in comparison to those suffering solely from asthma or COPD [13–20]. Due to these features, its significance has been highlighted as one of an etiotype of COPD, referred to as COPD & asthma (COPD-A), in the recently updated Global Initiative for COPD report [21]. However, data regarding the impact of PM exposure on ACO patients are very limited. This limitation is primarily attributed to the lack of suitable ACO experimental models.

We have previously developed two types of murine models by either intraperitoneal injection of ovalbumin (OVA)/aluminum hydroxide (alum) and intratracheal administration of porcine pancreatic elastase (PPE, ACO-a model) or intratracheal administration of papain (ACO-b model) that reflect the heterogeneity of ACO [22]. Clinical relevance of these two models has been validated based on changes in cytokine profile and lung function parameters compared to asthma and COPD models. However, the ACO model had a limitation in that it did not incorporate smoking as an additional risk factor. Therefore, this study aimed to set up experimental COPD models incorporating exposure to smoking and to investigate the harmful effects induced by PM differed between the COPD etiotype models.

## Materials and methods

### Mice and model establishment

In this investigation, 8-week-old female C57BL/6 N mice, obtained from Orient in Gyeongi-do, Korea, were utilized. These mice were randomly allocated into three distinct groups: control, cigarette smoking-related COPD (COPD-C), and COPD-A.

The COPD-C model was achieved by administering 80 U/kg of Porcine Pancreatic Elastase (PPE, Elastin Products Company, Owensville, MI, USA) in 50 µL of Phosphate Buffered Saline (PBS) through intratracheal (i.t.) instillation using a microsprayer aerosolizer from Penn Century Inc., located in Wyndmoor, PA, USA on day 0. Additionally, 50 µL of Cigarette Smoke Extract (CSE) was introduced intranasally (i.n.) to further mimic COPD-like conditions.

**Fig. 1:**
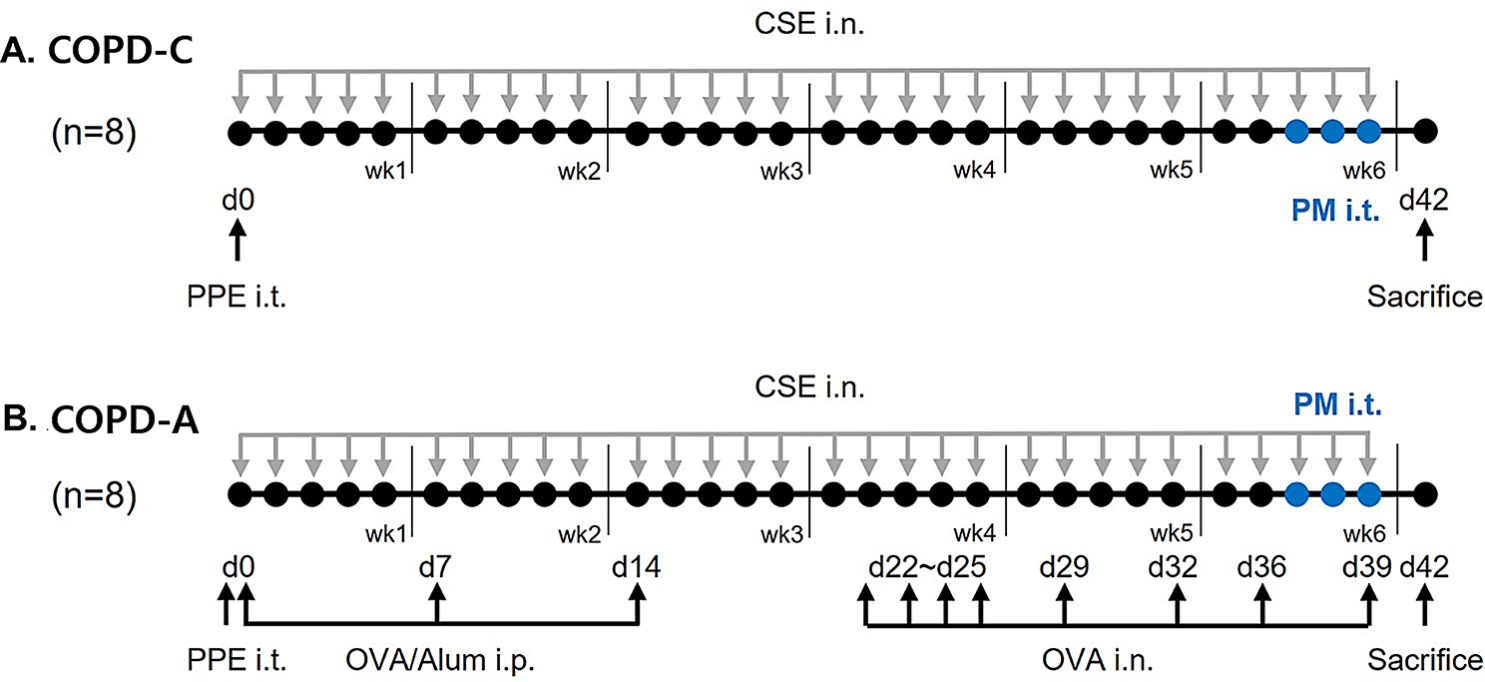
Scheme of (**A**) COPD-C and (**B**) COPD-A model construction and experimental workflow.COPD, chronic obstructive pulmonary disease; alum, aluminum hydroxide; CSE, cigarette smoke extract; i.n., intranasal; i.p., intraperitoneal; i.t., intratracheal; OVA, ovalbumin; PM, particulate matter; PPE, porcine pancreatic elastase

The COPD-A model was established by administering 80 U/kg of PPE in 50 µL of PBS intratracheal instillation using a micro sprayer aerosolizer on day 0. Additionally, to induce asthmatic features, 50 µg of Ovalbumin (OVA, Sigma-Aldrich, St. Louis, MO, USA) and 1 mg of aluminum hydroxide (Sigma-Aldrich) in 100 µL of PBS were injected intraperitoneally (i.p.) on days 0, 7, and 14. OVA at a concentration of 100 µg in 50 µL of PBS was intranasally given to the subjects on days 22, 23, 24, 25, 29, 32, 36, and 39, with isoflurane anesthesia provided by Vedco, located in St. Joseph, MO, USA. Additionally, to stimulate COPD features, 50 µL of CSE was also administered intranasally. Before instillation (i.t. and i.n.), mice were lightly anesthetized with isoflurane (Vedco, St. Joseph, MO, USA). Animals were humanely sacrificed on the 25^th^ day. The experiment scheme is illustrated in Fig. 1.

For the Control group, administration of PBS (i.t) was carried out in the same manner to the PM treatment process.

### Urban PM

The Urban PM used in the study was sourced from the National Institute of Standards and Technology, specifically the SRM 1648a standard. This PM10 reference material exhibited an average particle size of 5.85 micrometers. For preparation, it underwent a dispersion process in water, utilizing ultrasonication for a duration of 10 min [23]. PM was suspended in PBS at a final concentration at 100 µg/50 µL. PM was used for treatment on days 36, 37, and 38.

### Preparation of cigarette smoke extract (CSE)

Market-available cigarettes (THIS brand, 84 mm in length and 8 mm in diameter, distributed by KT&G, based in Seoul, Korea) underwent a consistent smoking process through a silicone tubing setup linked to a Variable-Flow Peristaltic Pump (manufactured by Fisherbrand in Shanghai, China). The smoke generated from a single cigarette was directed through a volume of 10 mL of PBS, at a flow rate of 50 mL per minute, sustained for a duration of 6 min [24]. Insoluble particles in the resulting suspension were filtered with a 0.22-µm filter.

### Bronchoalveolar lavage fluid (BALF) collection

Bronchoalveolar lavage fluid (BALF) was collected after sacrifice under anesthetizing with an intraperitoneal injection of a mixture of Rompun and Zoletil (1:4). Methods for the BAL procedure and specimen processing were performed according to a previous experiment and are described in detail in S4.

### Cytokine assay by enzyme-linked immunosorbent assay

Concentrations of neutrophil gelatinase–associated lipocalin (NGAL) in both serum and BALF and concentrations of various cytokines including interleukin (IL)-4, IL-13, IL-6, and tumor necrosis factor-α (TNF-α) in BALF were measured using ELISA kits (R&D Systems, Minneapolis, MN, USA) according to the manufacturer’s instructions.

### Lung histopathology

For scoring airway inflammation, lung tissue sample slides were numbered randomly and evaluated independently by two blinded investigators. The quantity of peribronchial or perivascular inflammation was assessed as described previously [25]. The calculation of the Mean Linear Intercept (MLI) involved the measurement of alveolar diameters across ten randomly selected fields on each slide, employing a Pannoramic MIDI slide scanner from 3DHISTECH Ltd., based in Budapest, Hungary, following previously established methods [26]. Detailed methods for lung samples processing methods were described in S6.

### ROS measurement

The quantification of cellular reactive oxygen species (ROS) was carried out utilizing dihydroethidium (DHE) obtained from Invitrogen, located in Carlsbad, CA, USA. For visual documentation, each slide was imaged using the LSM 900 confocal laser scanning microscope, a product of Carl Zeiss based in Jena, Germany. Further methods for detecting ROS via DHE in lung tissue are detailed in S7.

### Real-time polymerase chain reaction

Total RNAs were isolated from lung homogenates using TRIzol reagent (Invitrogen, Grand Island, NY, USA) and reverse-transcribed. Real-time PCR was performed using a CFX96 Real-Time PCR Detection System (Bio-Rad Laboratories, Hercules, CA, USA). Primers for PCR are listed in Table S1. The amplification process employed the iQ SYBR gene expression assay kit provided by Bio-Rad Laboratories, adhering to the guidelines specified by the manufacturer.

### Western blot analysis

Antibodies against caspase-3, B-cell lymphoma protein 2 (Bcl-2), Bcl-2 associated X (Bax) from Cell Signaling Technology (Beverly, MA, USA), and transcription factor nuclear factor erythroid-derived 2-like 2 (Nrf-2) were purchased from Novous Biologicals (Centennial, CO, USA) and analyzed by Western blot. Detailed methods for Western blot analysis were described in S9.

### Statistical analysis

The Data are expressed as the mean ± standard error of the mean (SEM). Group comparisons were conducted using one-way analysis of variance (ANOVA) followed by a Tukey post hoc test or two-way ANOVA coupled with the Bonferroni correction, utilizing the GraphPad Prism software (GraphPad Software, Inc., San Diego, CA, USA). Statistical significance was established at *P*-values of less than 0.05.

## Results

### BALF cell count and leukocyte composition

**Fig. 2:**
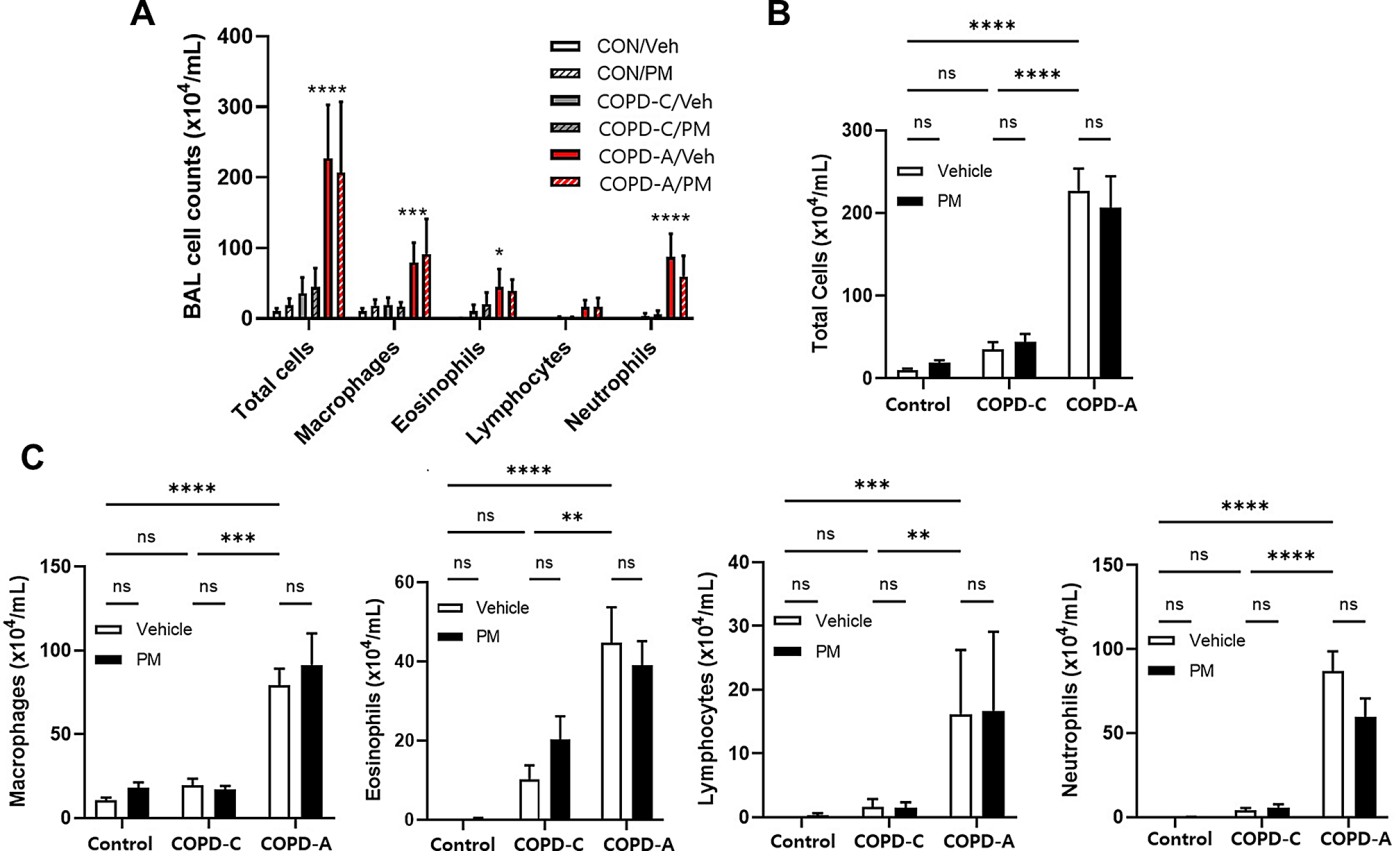
Differential cell counts in bronchoalveolar lavage fluid (BALF). The distribution of cell counts in BAL fluid is depicted in (**A**), the change in total cell counts is presented in (**B**), and the alterations in COPD-C and COPD-A model according to PM treatment for each cell type is illustrated in (**C**). COPD, chronic obstructive pulmonary disease; PM, particulate matter. *: *p* < 0.05, **: *p* < 0.01, ***: *p* < 0.001, ****: *p* < 0.0001, and ns: non-significant

Figure 2 displays the composition of inflammatory cells in BALF for each COPD model and the changes in cells due to PM exposure. In COPD-A model, there was a marked elevation in the total cells count, encompassing macrophages, eosinophils, and neutrophils, in comparison to the levels observed in both the control group and the COPD-C model. However, treatment with PM did not lead to a pronounced increase or change in the composition of inflammatory cell counts in BALF across all airway disease models, including COPD-A.

### Inflammatory cytokines Associated with Chronic Airway Disease

**Fig. 3:**
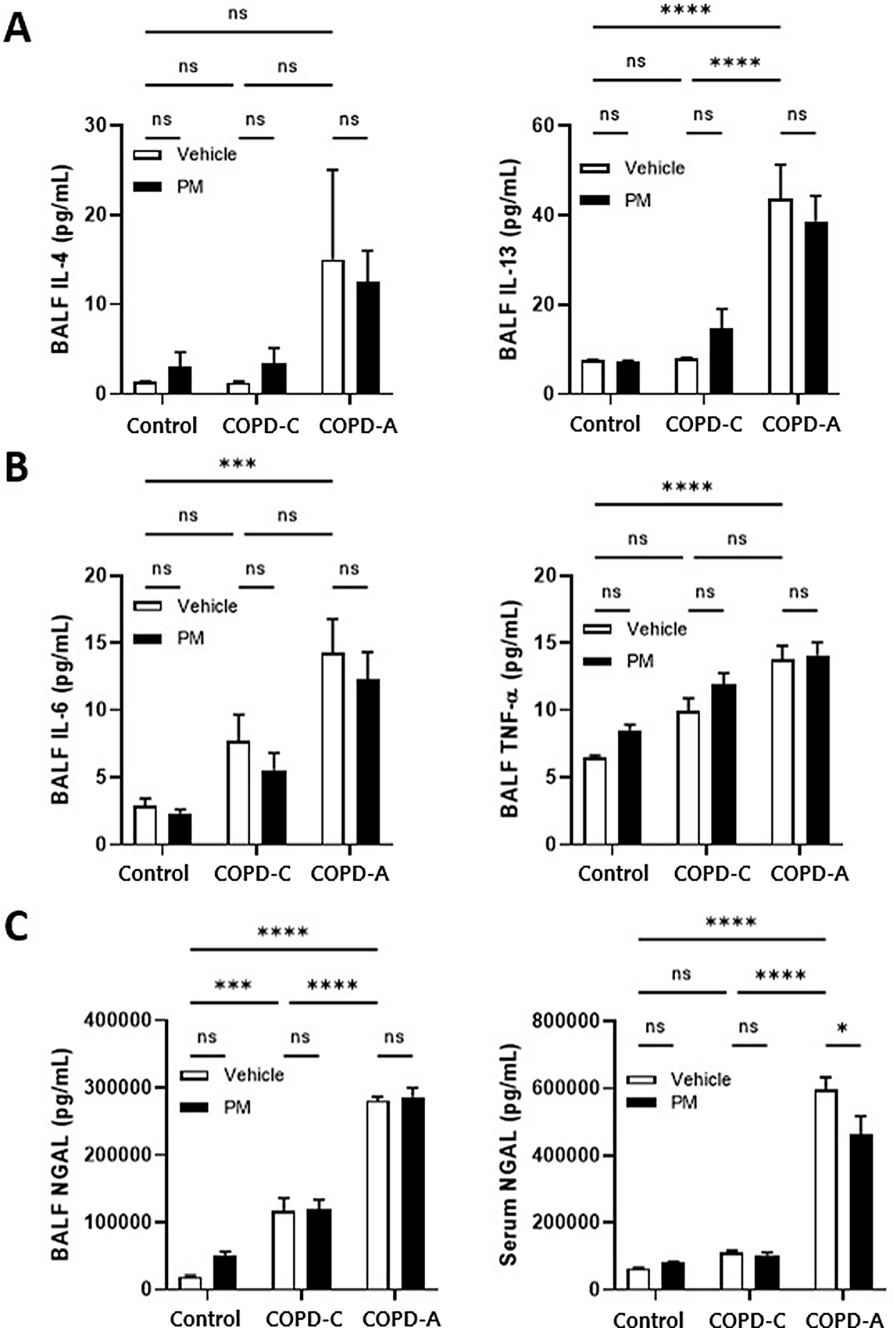
Inflammatory cytokines. (**A**) Type-2 inflammatory cytokines in BALF, (**B**) Macrophage/neutrophil-associated inflammatory cytokines in BALF, and (C) NGAL in BALF and serum. COPD, chronic obstructive pulmonary disease; BALF, bronchoalveolar lavage fluid; IL, interleukin; NGAL, neutrophil gelatinase–associated lipocalin; PM, particulate matter; TNF-α, tumor necrosis factor-α. *: *p* < 0.05, ***: *p* < 0.001, ****: *p* < 0.0001, ns: non-significant

We employed ELISA to quantitatively assess the levels of inflammatory cytokines in BALF. The results indicated a heightened presence of type 2 inflammatory cytokines, specifically IL-4 and IL-13, in the COPD-A model relative to the levels in both the control group and COPD-C model. However, exposure to PM did not induce significant changes in these type 2 inflammatory cytokine levels (Fig. 3A).

Cytokines associated with neutrophilic inflammation, such as IL-6 and TNF-α, exhibited an increasing trend in both COPD models compared to the control group. Moreover, TNF-α exhibited a tendency to increase following PM exposure in all models, albeit without reaching statistical significance (Fig. 3B).

Levels of NGAL in BALF and serum were quantified (Fig. 3C). The concentration of NGAL in BALF was elevated in COPD relative to control group, with an additional increase observed in the COPD-A model. In both COPD models, NGAL levels remained unchanged following PM treatment. Conversely, in the control group, a rise in NGAL levels was noted after PM treatment, although this difference did not achieve statistical significance. NGAL levels in serum were significantly higher in the COPD-A model compared to both the control and COPD-C models. Furthermore, PM treatment resulted in a significant reduction of NGAL levels within the COPD-A model.

### Airway inflammation and airspace enlargement

**Fig. 4:**
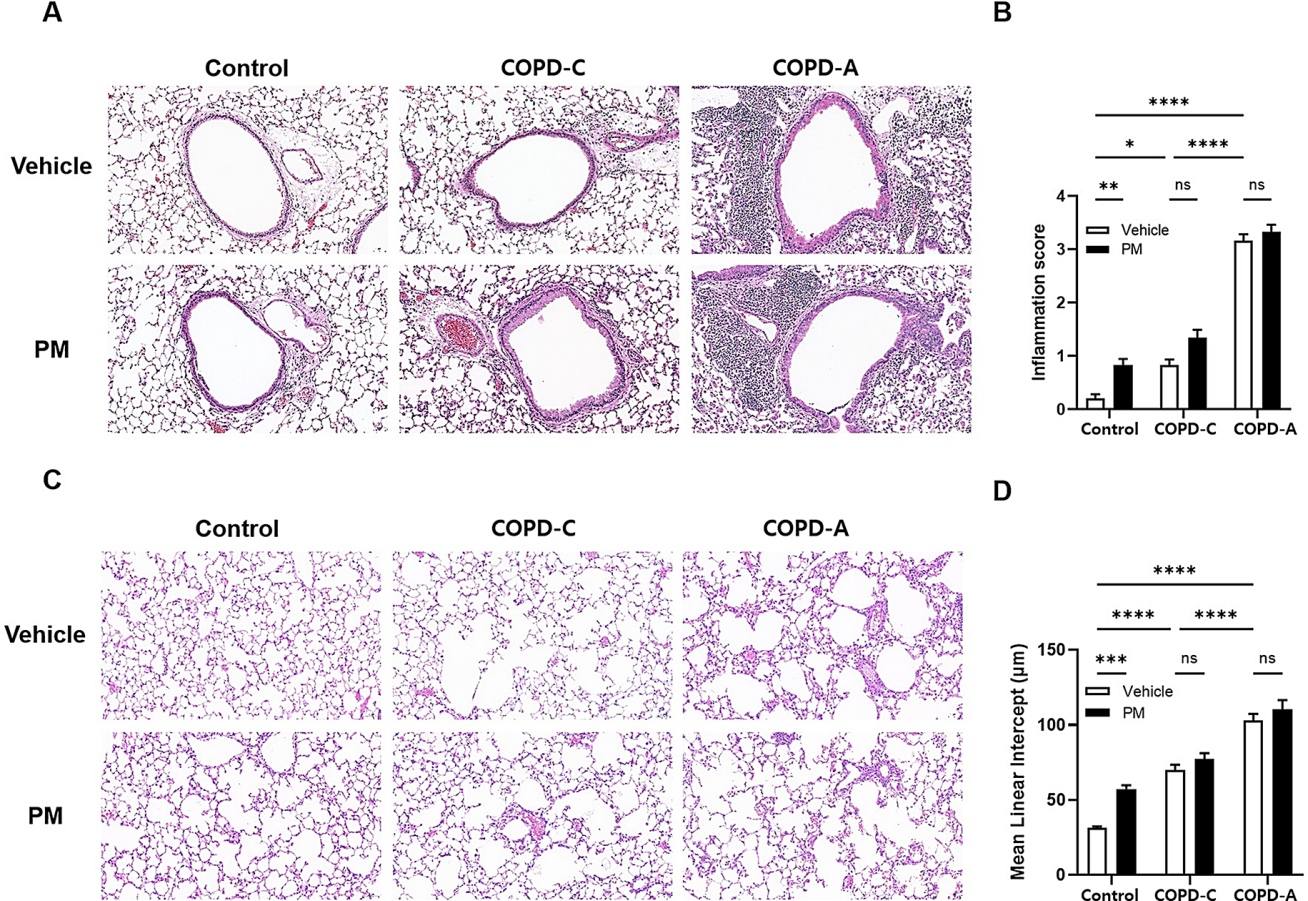
Histopathological changes in airway inflammation and airspace in different airway models by PM. (**A**) Hematoxylin–eosin (H&E) staining of right lung sections from COPD-C and COPD-A mice. Bright-field microscopy images show inflammatory responses around airways, (**B**) Inflammation grades for peribronchial and perivascular inflammation from H&E staining, (**C**) Lung parenchyma stained with H&E showing airspace enlargement, and (**D**) Airspace size evaluated by the mean lineal intercept. COPD, chronic obstructive pulmonary disease; PM, particulate matter. *: *p* < 0.05, **: *p* < 0.01, ***: *p* < 0.001 and ****: *p* < 0.0001, and ns: non-significant

Lung histology was examined through the application of H&E staining across the three different models (Fig. 4A). Relative to the control group, both COPD models demonstrated elevated inflammation scores, predominantly due to the intrinsic nature of the disease. Nevertheless, no marked difference in inflammation scores was observed following PM exposure within these disease categories. Interestingly, in the control group, the inflammation score experienced a significant rise following treatment with PM (Fig. 4B). Our investigation revealed a significant increase in airspace enlargement in both COPD models compared to the control group (Fig. 4C). While the impact of PM exposure was not pronounced in underlying chronic pulmonary disease models, COPD-C and COPD-A, it was noteworthy that exposure to PM resulted in a significant increase in airspace enlargement in the control group (Fig. 4D).

### Apoptosis and related protein expression

**Fig. 5:**
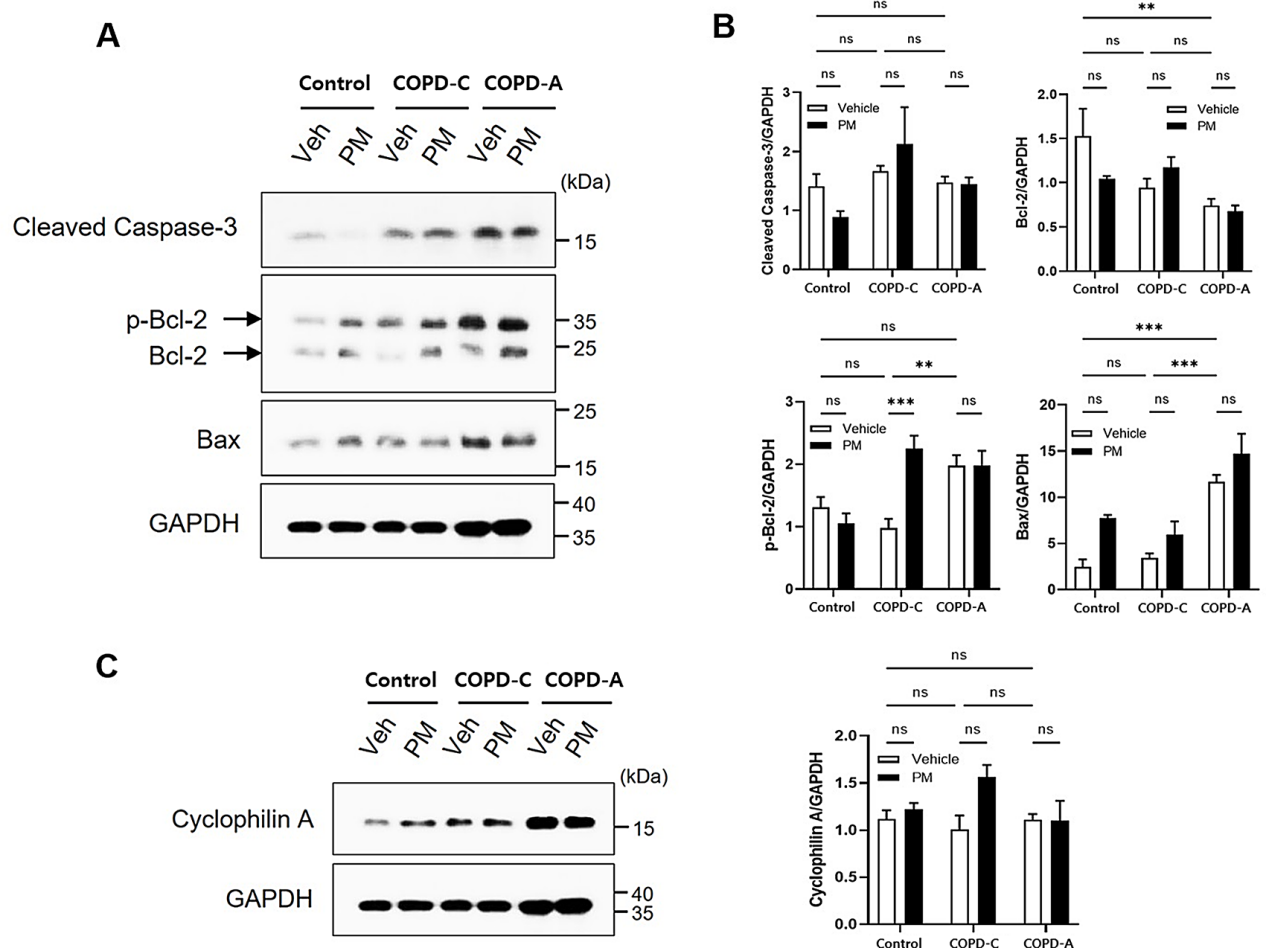
Cell damage mechanisms. (**A**) Western blot analysis was performed to detect expression of apoptosis related proteins in lung tissues from each group with GAPDH as a loading control, (**B**) Results of densitometry analysis. Protein levels of cleaved caspase-3, Bcl-2, phosphorylated Bcl-2, and Bax were quantified and normalized to GAPDH band intensity, (**C**) Necrotic protein cyclophilin A expression was assessed and quantified. Bcl-2, B-cell lymphoma protein 2; Bax, Bcl-2 associated X; GAPDH, glyceraldehyde-3-phosphate dehydrogenase; PM, particulate matter. **: *p* < 0.01 and ***: *p* < 0.001, ns: non-significant

The expression of proteins associated with cellular damage mechanisms in each airway disease model was investigated using western blot analysis (Fig. 5). Caspase 3 exhibited no significant differences among control, COPD-C, and COPD-A groups. It showed no significant changes following exposure to PM either (Fig. 5A). Bcl-2 displayed a decreasing tendency in both COPD models compared to the control, with PM exposure having no significant impact on Bcl-2 expression. However, p-Bcl-2 level was increased in COPD-A compared to that in the control. It was significantly increased in COPD-C following PM exposure. Bax protein expression exhibited a progressive increase from the control group to both COPD models. Although its increases did not demonstrate a statistical significance, it consistently displayed an upward trend across all three groups following PM treatment (Fig. 5B).

In the assessment of alternative cell death pathways, expression of cyclophilin A protein as a marker associated with necrosis was analyzed. However, no discernible alterations were observed in relation to PM exposure. Similarly, no distinctions were evident in the airway disease model employed (Fig. 5C). LC3B expression was also analyzed to appraise autophagy activity. It showed no significant disparities upon PM exposure or in specific airway disease models (data not shown).

### Oxidative stress

**Fig. 6:**
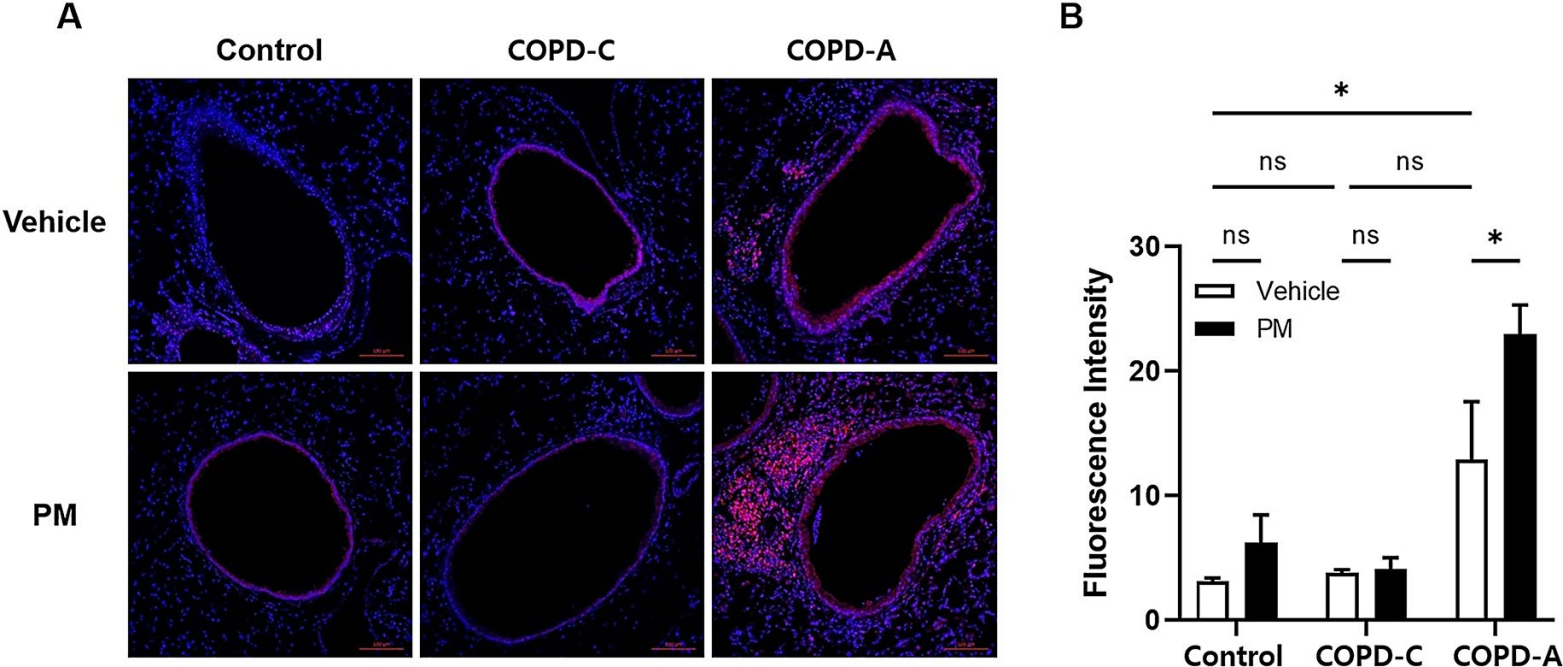
Production of ROS. (**A**) Tissue sections of small airways and alveoli were stained with DHE (pink) and nuclear staining was done with DAPI (blue), (**B**) Fluorescence intensity of DHE from each group. Bars represent the mean ± SEM of results (*n* = 6). COPD, chronic obstructive pulmonary disease; DHE, dihydroethidium; ROS, reactive oxygen species; PM, particulate matter. *: *p* < 0.05, ns: non-significant

The study conducted a thorough investigation into the magnitude of ROS expression levels. Cells exhibiting bright fluorescent red in their nuclei indicative of ROS-positive were higher in the COPD-A model than in the control and COPD-C (Fig. 6A). Moreover, the intensity of ROS expression was increased in the only COPD-A model after PM treatment (Fig. 6B).

**Fig. 7:**
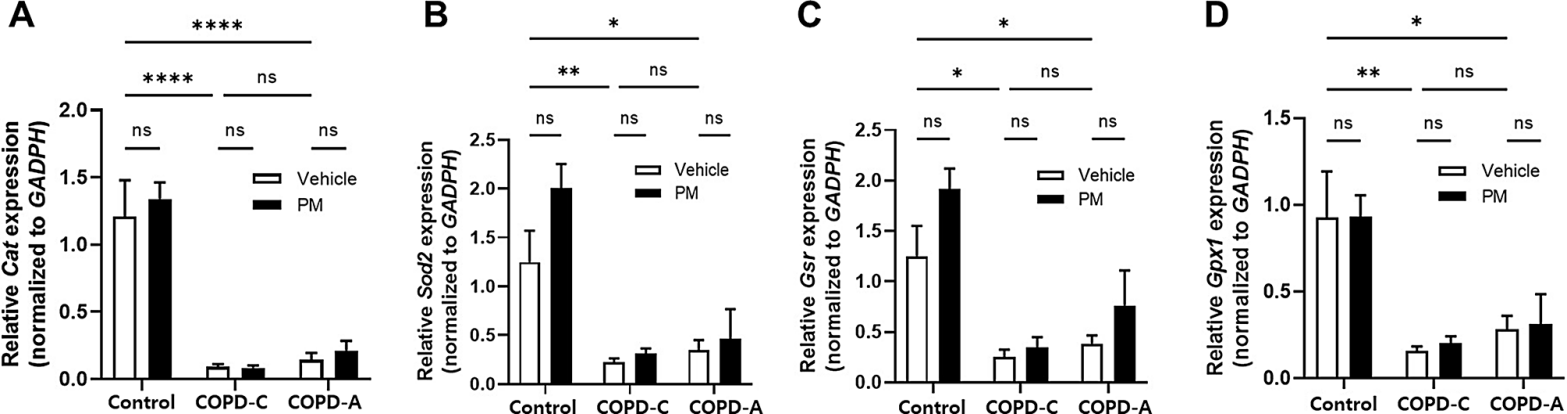
Relative expression of genes encoding ROS-detoxifying enzymes. RT-qPCR analysis of differentially expressed ROS-related genes, (**A**) Cat, (**B**) Sod2, (**C**) Gsr, and (**D**) Gpx1. Expression of each enzyme level was quantified and normalized to GAPDH. COPD, chronic obstructive pulmonary disease; Cat, catalase; GAPDH, glyceraldehyde-3-phosphate dehydrogenase; Gpx, glutathione peroxidase; Gsr, glutathione reductase; PM, particulate matter; ROS, reactive oxygen species; Sod2, superoxide dismutase. *: *p* < 0.05, **: *p* < 0.01, and ****: *p* < 0.0001, ns: non-significant

We assessed antioxidant enzymes involved in the protection against oxidative stress using RT-qPCR. In both COPD models, expression levels of enzymes such as Cat, SOD2, GSR, and GPX1 were decreased compared to those in the control. However, PM exposure had no significant effect on the expression of these antioxidant enzymes in any airway disease models (Fig. 7).

**Fig. 8:**
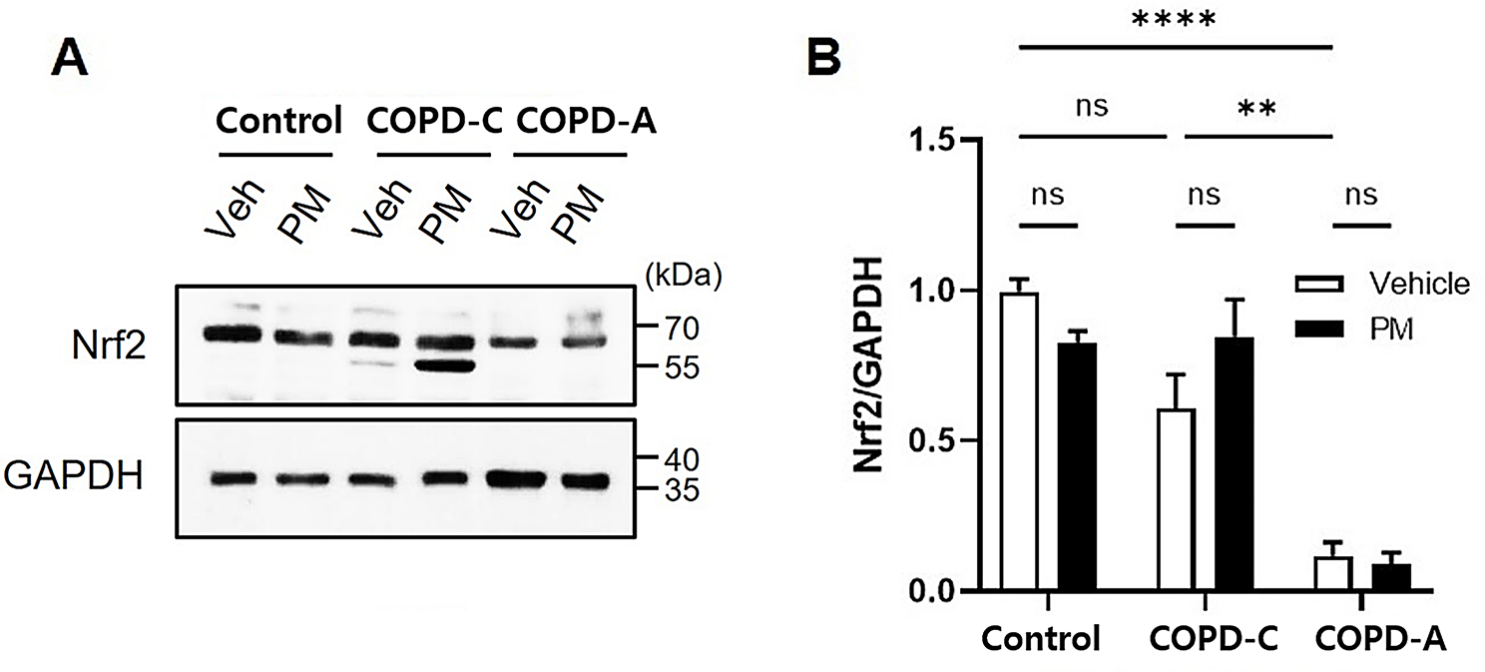
Difference in Nrf-2 expression in airway models and impact of PM. (**A**) Expression of Nrf-2 was investigated by Western Blot analysis and GAPDH was used as a loading control, (**B**) Nrf-2 protein level was quantified and normalized to GAPDH band intensity. COPD, chronic obstructive pulmonary disease; GAPDH, glyceraldehyde-3-phosphate dehydrogenase; Nrf-2, nuclear factor erythroid-derived 2-like 2; PM, particulate matter. **: *p* < 0.01, and ****: *p* < 0.0001, ns: non-significant

The expression of Nrf-2 known to play a protective role against oxidative stress triggered by inflammation or injury [27, 28] was investigated across all airway disease models. Compared to the control and COPD-C, COPD-A exhibited a significant decrease in baseline Nrf-2 expression (Fig. 8). However, PM treatment did not result in any significant change in Nrf-2 expression. Despite the absence of statistical significance, PM treatment resulted in a modest elevation of Nrf-2 expression in the COPD-C model and an additional Nrf-2 band was unexpectedly detected in Western blot analysis (Fig. 8A).

## Discussion

COPD is regarded as a preventable, treatable pulmonary disease and is a heterogeneous disease caused by various factors [21] While the harmful effects of PM on COPD have been widely reported, studies examining differences based on etiotypes are extremely rare. In this experimental study, we constructed two etiotypes corresponding to COPD-C, which is accompanied by emphysema, and a COPD-A model. Then we compared the effects of PM exposure on both models. Analyzing the impact of short-term PM exposure on normal, COPD-C, and COPD-A models demonstrated obvious susceptibility to oxidative stress and higher expression of apoptosis related protein in the COPD-A model compared to others. Moreover, we found evidence of development of COPD upon PM exposure in normal control by increasing airway inflammation and emphysema. This is an interesting observation aligned with the concept of biomass and pollution exposure COPD (COPD-P) [21], as air pollution can act as a risk factor not only for the exacerbation and progression of COPD but also for the development of the disease itself. This could expand our insight into cell damage and difference in susceptibility to PM exposure between normal and different etiotypes of COPD.

Noxious effects of PM on chronic airway disease such as asthma and COPD are well-understood as evidenced by many epidemiological studies. A comprehensive meta-analysis has revealed a positive correlation between asthma incidence in children and teens and exposure to air pollution from traffic sources [29]. These findings collectively underscore the significant impact of PM on respiratory health, particularly in vulnerable populations such as children. A particular prospective research study has underscored the link between exposure to PM during pregnancy and an increased likelihood of childhood asthma development [30]. In the context of COPD, an increasing level of PM exposure not only amplifies morbidity by exacerbating pre-existing COPD conditions [31–33], but also increases the incidence of COPD through meta-analysis [34]. Study from Canada have suggested a higher risk of transitioning to ACO with increased air pollution exposure in asthma [35]. One study based on the Korean National Health Insurance Service database has reported higher mortality in ACO due to PM_10_ exposure than in pure COPD [36] but lack of information regarding impact of PM on ACO.

Unlike more common chronic airway diseases such as asthma and COPD, the impact of air pollution on ACO remains relatively understudied mainly attributed to difficulty in defining ACO patients and in establishing an animal model. In our recent research, we have developed two types of ACO models, each embodying characteristics of both asthma and COPD. The adequacy of these ACO models was validated through comparisons with the individual models for asthma and COPD [22]. To discern the underlying mechanisms of distinctions between COPD and ACO, comprehensive mouse models were employed by adding an impact of cigarette smoking. The ACO model demonstrated elevated levels of pro-inflammatory mediators, indicative of both type 2 and neutrophilic inflammation. Whole-body CS exposure better mimics real-world smoking and induces more severe inflammation due to higher serum continine levels [37]. However, CSE exposure also triggers significant neutrophilic influx and protein kinase C activity [38], with both methods resulting in similar lung function impairment [37, 39]. Additionally, CSE offers more precise control and measurement of exposure. Our previous research, along with studies from others, supports the validity of using CSE in COPD modeling [24, 40, 41]. This approach allowed us to develop aa more insightful ACO model that accurately reflects its distinct pathophysiology from COPD, while complementing the limitations of our previous ACO model.

Mechanism of PM-induced cell damage has not been clearly revealed yet. It is known to be multifactorial. We approached it via three pathways to PM related cell death and found that necrosis and autophagy pathway were not significantly affected by PM exposure in any models. However, expression levels of apoptosis related protein were affected by PM treatment. Furthermore, the production of ROS associated with oxidative stress was notably greater in the COPD-A model than in both the control group and COPD-C model. In addition, we assessed mechanism relating ROS generation and found that Nrf-2 expression was much lower in the COPD-A model. Nrf-2, a transcription factor, can regulate the expression of antioxidant proteins and protect against oxidative damage triggered injury and inflammation [27, 28]. In bronchial epithelial cells, PM treatment can reduce Nrf-2 expression and curcumin, a natural component extracted from plant *Curcuma longa* with anti-inflammatory and anti-oxidant activities [42, 43], can prevent PM-induced Nrf-2 suppression [44, 45]. In this study, both COPD models had lower Nrf-2 expression compared to the control. PM treatment did not further reduce the Nrf-2 level. Moreover, expression levels of antioxidant enzymes such as SOD, catalase, Gsr, and Gpx were found to be further reduced in both COPD models when compared with the control group. In addition, PM treatment did not show any further decrease in expression level of these enzymes. We assumed that Nrf-2 mediated antioxidant response system in lung tissues was much disturbed in disease models, especially in the COPD-A model. Such disturbance of redox homeostasis in lungs was expressed by elevating ROS levels in the COPD-A model.

An important outcome of this experiment was that we found distinctions between COPD-C and COPD-A in pathophysiology as well as harmful effects of PM in each model. Under a stressful condition, keap1, a regulatory protein that binds to Nrf-2 in the cytoplasm, is deactivated. Stabilized Nrf-2 undergoes nuclear translocation and leads to cell protection through the antioxidant response element, consequently activating target genes [46]. In a PPF-induced COPD model, Nrf-2 knockout mice exhibited heightened sensitivity to COPD compared to wild type mice [47]. The current study observed very low Nrf-2 expression in COPD-A, showing no significant decrease after PM exposure. However, in the context of COPD-C, PM exposure resulted in a slight increase in Nrf-2 expression and additional bands were unexpectedly detected. Considering concurrent increases of emphysema and airway inflammation with an increase of ROS, it can be postulated that a slight increase in Nrf-2 expression may not necessarily denote a functional increase. These findings imply potential differences in oxidative stress response mechanisms between COPD-C and COPD-A.

Another noteworthy outcome of this study was that there were changes due to PM exposure in the control model. Large-scale, long-term epidemiologic studies have consistently indicated a more pronounced decrease in forced expiratory volume in 1 s (FEV_1_) with higher levels of PM exposure [48, 49]. Zhao et al. [50] have reported a synergistic impact of PM2.5 and cigarette smoke on COPD development and progression in both in vivo and in vitro studies. They found that inflammation and emphysematous changes were the underlying mechanisms. Additionally, it has been observed that reduced PM exposure can lead to a mitigation in the decline rate of lung function, particularly in small airway function [51]. The mechanisms underlying the impact of PM on the respiratory system involve oxidative stress, mitochondrial damage, inflammation of airway epithelial cells, cell damage, extracellular matrix remodeling, and fibrosis [7]. Consistent with results of previous studies, our findings demonstrated that significantly more severe inflammation and emphysematous changes were developed by PM exposure in normal controls. This provides broader insight to the pathophysiology of PM-induced chronic airway diseases.

We successfully developed a highly reproducible model of distinct COPD etiotypes. Through these models and comparison with the control group, we gained valuable insights into the varying susceptibility to cell damage across different COPD models, particularly apoptosis pathway and an increase in ROS during the cell damage process induced by exposure to PM. This is the principal strength of this study. However, it is important to acknowledge several limitations inherent in this research. Firstly, to enhance the feasibility of modeling airway diseases, we employed CSE intranasal instillation throughout the entire experimental period, complemented by PM intratracheal treatment administered over the final three days. This short-term exposure regimen primarily captured PM-related early-onset inflammation, precluding the observation of effects associated with long-term PM exposure. Secondly, with respect to oxidative stress, our meticulously crafted COPD-C and COPD-A models exhibited a reduced antioxidant capacity attributed to the inherent nature of these diseases even before PM treatment. This diminished baseline antioxidant defense made it challenging to identify a specific protective target. Subsequent investigations essential to evaluate the long-term stability and reproducibility of PM exposure models. Further exploration and future studies are warranted to delineate effective strategies and potential targets for mitigating oxidative stress in the context of chronic airway diseases. Thirdly, both COPD models were developed over a 6-week period, a duration thought to be comparable to that of the acute asthma model. It has been reported that PM-induced worsening of asthma occurs through largely common mechanisms in both acute and chronic asthma models [52–55]. However, since this study focused on the differences between COPD etiotypes, a direct comparison with the asthma model for the same period of exposure was not performed.

## Conclusion

We developed representative COPD and ACO models, which correspond to two different etiotypes of COPD. These models, known for their simplicity and ease to setup, also exhibit a notable degree of reproducibility. After short period of PM treatment, increased expression of apoptosis related proteins and increased oxidative stress in COPD-A model were found. In addition, PM treatment substantially contributed to the development of pathologic features of COPD in normal controls, aligning with the concept of COPD caused by biomass and pollution exposure. Future studies with long-term PM exposure are needed to investigate real-world air pollution related respiratory disease.

## Electronic supplementary material

Below is the link to the electronic supplementary material.

Supplementary Material 1

Supplementary Material 2

## Data Availability

No datasets were generated or analysed during the current study.

## Abbreviations

BALF: Bronchoalveolar lavage fluid
Bcl-2: B-cell lymphoma protein 2
Bax: Bcl-2 associated X
ACO: Asthma and chronic obstructive pulmonary disease (COPD)
Cat: Catalase
CSE: Cigarette smoke extract
GAPDH: Glyceraldehyde-3-phosphate dehydrogenase
Gpx: Glutathione peroxidase
Gsr: Glutathione reductase
IL: interleukin
MLI: Mean linear intercept
NGAL: Neutrophil gelatinase–associated lipocalin
OVA: Ovalbumin
PM: Particulate matter
PPE: Porcine pancreatic elastase
ROS: Reactive oxygen species
Sod2: Superoxide dismutase
TNF-α: Tumor necrosis factor-α
