# Supplementary material for: Influence and distinctions of particulate matter exposure across varying etiotypes in chronic obstructive pulmonary disease (COPD) mouse model
Source: J Inflamm (Lond). 2024 Nov 1;21:42. doi: 10.1186/s12950-024-00416-8 (PMC11529024; doi:10.1186/s12950-024-00416-8)
Supplement: Supplementary file 1 — Supplementary Material 1 [file 12950_2024_416_MOESM1_ESM.docx]

**"Online Supplementary Methods"**

**Influence and distinctions of particulate matter exposure across varying etiotypes in chronic obstructive pulmonary disease (COPD) mouse model**

S4 Methods for BAL and specimen processing

The exposed trachea was cannulated with silicone tubing attached to a 22-guage needle on a 1-mL tuberculin syringe. BALF was collected after instillation of 0.8 mL sterile PBS and centrifuged at 3,000 rpm for 5 min at 4℃. Supernatants were collected and stored at –80℃. Total cell counts in BALF were obtained using a LUNA^TM^ automated cell counter (Logos Biosystems, Inc., Annandale, VA, USA). The BALF was centrifuged onto microscope slides at 2,000 rpm for 7 min in a Cytospin centrifuge (Thermo Fisher Scientific, Waltham, MA, USA) and stained with a Shandon Kwik-Diff™ kit (Thermo Fisher Scientific). Macrophages, eosinophils, lymphocytes, and neutrophils in BALF were enumerated by counting 500 leukocytes in randomly selected fields under a light microscope.

S6. Methods for lung samples processing methods

Lung samples were fixed in 4% paraformaldehyde and embedded in paraffin wax. Sections were cut to 4 μm in thickness using a microtome. Deparaffinized tissue sections were stained with hematoxylin and eosin (H&E) to detect cellular infiltration.

S7. Methods for detecting ROS in lung tissue

DHE would exhibit a pink fluorescence when oxidized. When DHE interacted with cell’s DNA, a bright fluorescent blue staining would appear. Paraffin-embedded lung tissue sections were deparaffinized and hydrated for DHE staining. After incubating tissue sections in 20 μM DHE for 1 h, 4', 6-diamidino-2-phenylindole (DAPI, Invitrogen, Carlsbad, CA, USA) was added and incubated for 10 min to detect nucleus. Stained tissues on slides were mounted with a mounting solution (Vector Laboratories, Burlingame, CA, USA). Images of each slide were taken using a confocal laser scanning microscope LSM 900 (Carl Zeiss, Jena, Germany).

S9. Methods for Western blot analysis

Lungs were extracted with RIPA buffer and centrifuged at 12,000 ×g for 10 minutes at 4°C. Protein contents in supernatants were quantified using BCA (Thermo Fisher Scientific, Rockford, IL, USA). Lung homogenates were then separated by 12% sodium dodecyl sulfate poly-acrylamide gel electrophoresis, transferred onto PVDF membranes (Millipore, Bedford, MA, USA), and blocked with 5% skim milk for 1 hour at room temperature. These membranes were blotted with monoclonal antibodies against specific proteins as indicated. Relative intensities of protein bands were quantified using the ImageJ software (National Institutes of Health, Bethesda, MD, USA).

**Table S1.** Primers used in the real-time quantitative PCR

| Enzyme | Forward primers (5’-3’) | Reverse primers (5’-3’) |
| --- | --- | --- |
| CAT | CGGCACATGAATGGCTATGGATC | AAGCCTTCCTGCCTCTCCAACA |
| SOD2 | TAACGCGCAGATCATGCAGCTG | AGGCTGAAGAGCGACCTGAGTT |

| GPX1 | CGCTCTTTACCTTCCTGCGGAA | AGTTCCAGGCAATGTCGTTGCG |
| --- | --- | --- |
| GSR | GTTTACCGCTCCACACATCCTG | GCTGAAAGAAGCCATCACTGGTG |
| GAPDH | GTGCTGAGTATGTCGTGGAG | ATTTCTCGTGGTTCACACCC |

CAT, catalase; GAPDH, glyceraldehyde-3-phosphate dehydrogenase; GPX1, glutathione peroxidase 1; GSR, glutathione reductase; SOD2, superoxide dismutase 2
