# Supplementary material for: Influence and distinctions of particulate matter exposure across varying etiotypes in chronic obstructive pulmonary disease (COPD) mouse model
Source: J Inflamm (Lond). 2024 Nov 1;21:42. doi: 10.1186/s12950-024-00416-8 (PMC11529024; doi:10.1186/s12950-024-00416-8)

**"Online Supplementary data of the Western blots"**

**Influence and distinctions of particulate matter exposure across varying etiotypes in chronic obstructive pulmonary disease (COPD) mouse model**

**Supplementary Figure 1.**  Uncropped and unedited versions Western blots of A) Figure 5A and B) Figure 5C


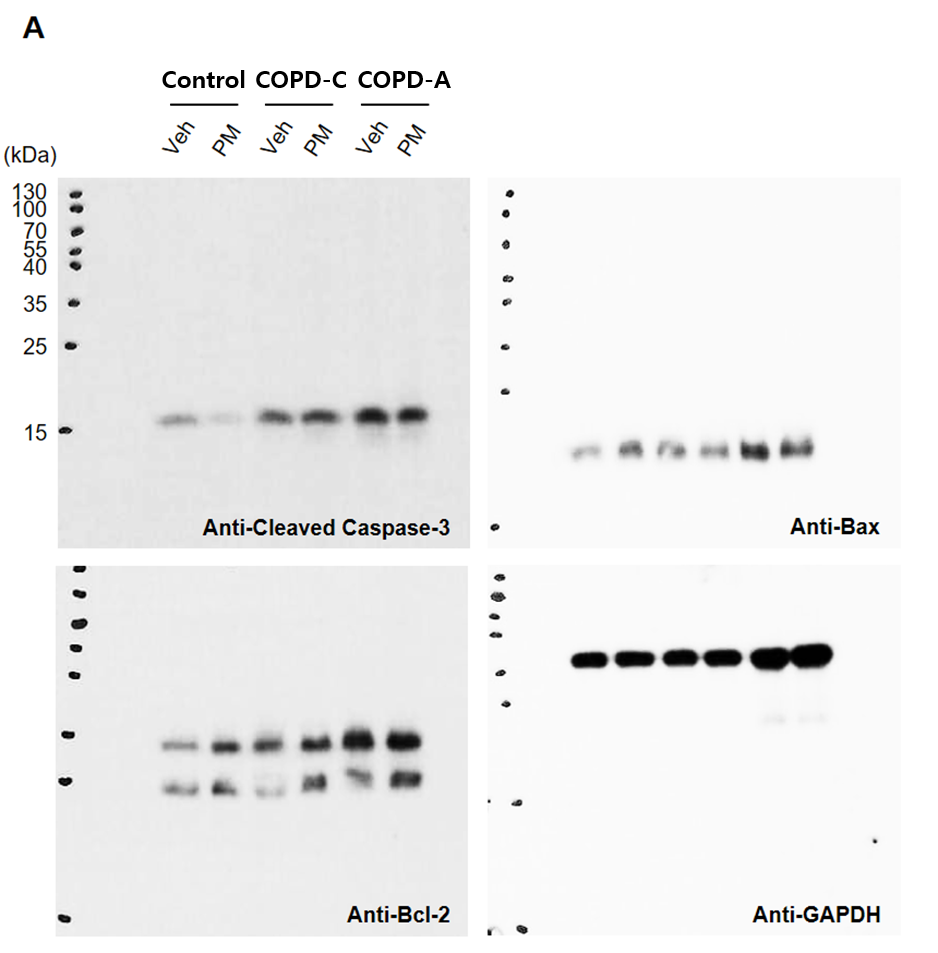


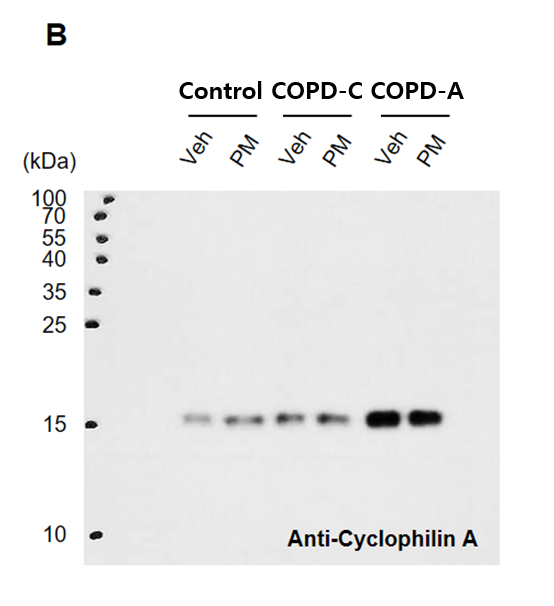


**Supplementary Figure 2.**  Uncropped and unedited versions Western blots of Figure 8A


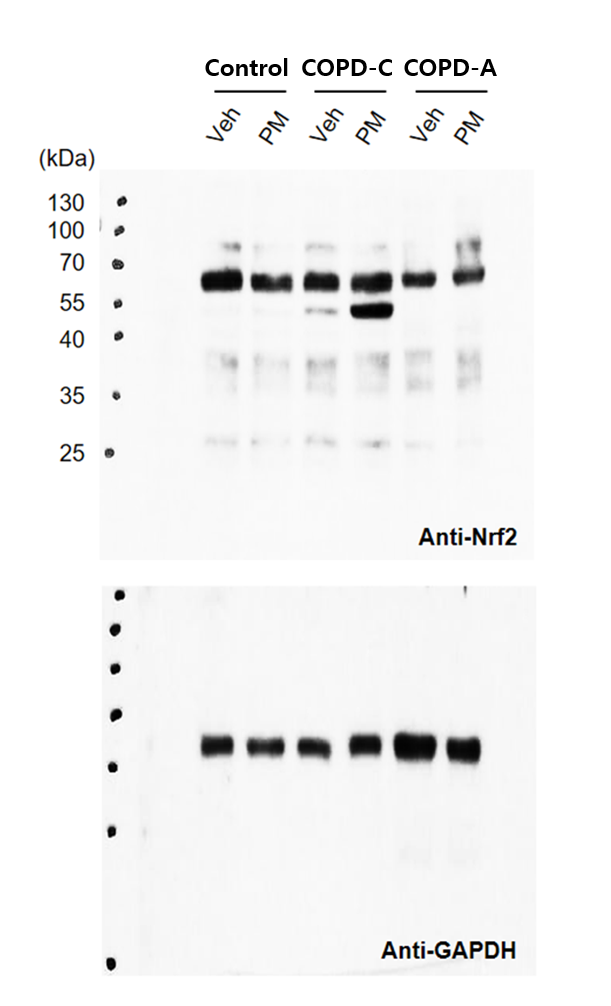

Supplement: Supplementary file 2 — Supplementary Material 2 [file 12950_2024_416_MOESM2_ESM.docx]
